# Sponge budding is a spatiotemporal morphological patterning process: Insights from synchrotron radiation-based x-ray microtomography into the asexual reproduction of *Tethya wilhelma*

**DOI:** 10.1186/1742-9994-6-19

**Published:** 2009-09-08

**Authors:** Jörg U Hammel, Julia Herzen, Felix Beckmann, Michael Nickel

**Affiliations:** 1 Institut für Spezielle Zoologie und Evolutionsbiologie, Friedrich-Schiller-Universität Jena, Erbertstr. 1, 07743 Jena, Germany; 2 GKSS Research Center, Max-Planck-Str. 1, 21502 Geesthacht, Germany

## Abstract

**Background:**

Primary agametic-asexual reproduction mechanisms such as budding and fission are present in all non-bilaterian and many bilaterian animal taxa and are likely to be metazoan ground pattern characters. Cnidarians display highly organized and regulated budding processes. In contrast, budding in poriferans was thought to be less specific and related to the general ability of this group to reorganize their tissues. Here we test the hypothesis of morphological pattern formation during sponge budding.

**Results:**

We investigated the budding process in *Tethya wilhelma *(Demospongiae) by applying 3D morphometrics to high resolution synchrotron radiation-based x-ray microtomography (SR-μCT) image data. We followed the morphogenesis of characteristic body structures and identified distinct morphological states which indeed reveal characteristic spatiotemporal morphological patterns in sponge bud development. We discovered the distribution of skeletal elements, canal system and sponge tissue to be based on a sequential series of distinct morphological states. Based on morphometric data we defined four typical bud stages. Once they have reached the final stage buds are released as fully functional juvenile sponges which are morphologically and functionally equivalent to adult specimens.

**Conclusion:**

Our results demonstrate that budding in demosponges is considerably more highly organized and regulated than previously assumed. Morphological pattern formation in asexual reproduction with underlying genetic regulation seems to have evolved early in metazoans and was likely part of the developmental program of the last common ancestor of all Metazoa (LCAM).

## Background

Sponges (Porifera) display a wide range of reproduction strategies, both sexual and asexual. Asexual reproduction in sponges occurs as a variety of mechanisms, including budding, fragmentation and gemmulation [1]. In general, asexual reproduction seems to be part of the ground pattern of all Metazoa [2]. However, primary agametic-asexual reproduction mechanisms such as fission or budding should be clearly distinguished from secondary asexual reproduction mechanisms such as parthenogenesis. Apart from in the poriferans, primary asexual reproduction is present in all non-bilaterian clades: Placozoa [3], Cnidaria [4] and Ctenophora [5], and even in most bilaterian clades [2], e.g. fission in Platyhelminthes. In addition, looking at Ediacaran fossils we find mainly colonial organisms, which also indicate that the ability to bud is fundamental to the metazoan condition [6]. Even though it has been suggested that much genomic repatterning occurred during Cambrian explosion [7], it seems likely that the regulation of agametic asexual reproduction in all these metazoans can be put down to a common early root, i.e. a set of genes which regulate pattern formation [8]. In the cnidarian model systems *Hydra *sp., *Nematostella vectensis *STEPHENSON, 1935 and *Podocoryna carnea *M. SARS, 1846, asexual reproduction follows strict temporal patterns which are genetically regulated [9]. Regulation of cnidarian budding involves developmental pathways, such as the *Wnt*-pathway [10], typically found in larval development. In contrast, asexual development in sponges is usually thought to be less specific and is interpreted as a consequence of the general ability of this group to reorganize constantly [11]. The high level of mobility of all cell types within the mesohyle of sponges (excluding the epithelial-like pinacocytes) is referred to as 'constant morphogenesis' [12]. Within the broader metazoan context the question arises as to whether poriferan asexual reproduction follows similar temporal patterns to those in cnidarians. If this is the case, asexual reproduction in sponges is most likely strictly regulated by developmental pathways.

In order to address this question it is useful to distinguish the processes in poriferans which are generally regarded as asexual reproduction. Fragmentation of specimens represents the most primitive mode. Ecologically and in the context of population dynamics, fragmentation maximizes dispersal and thus represents an important reproduction strategy which might exceed sexual reproduction rates in many species [1,13]. However, the main prerequisites for this mode of reproduction are the general morphological plasticity of sponges and their ability to reorganize. The literature does not point towards the involvement of highly regulated processes [14]. This is further supported by the fact that fragmentation has been shown to be linked to specific sexual reproduction events in some cases [15].

Gemmulation and the structure of gemmules in freshwater sponges has been studied intensively [16] and is an important part of the life history of several species. However, gemmules cannot be regarded as asexual reproduction bodies in the strict sense. In most cases, they serve as dormant structures that can be produced to overcome unfavorable environmental conditions. Gemmules are formed inside the sponge tissue in high numbers and remain embedded in the skeleton framework when the mother sponge decays. Although gemmules might be dispersed (which in fact represents asexual multiplication), they mainly repopulate the skeletal structures of the former mother sponge [16]. Gemmule formation and hatching thus represent what is presumably a highly derived case of sponge morphogenesis restricted to freshwater sponges (suborder Spongillina) and a few other families.

While both fragmentation and gemmulation cover a variety of purposes in the life history of some sponges, budding only fulfils one function. Bud formation, release and subsequent morphogenesis are exclusively processes of asexual reproduction. Although budding is only obligate in the life histories of the two families Polymastiidae and Tethyidae, it occurs on an irregular basis in most, if not all demosponge families [1,17].

Maas [18], and Connes [19] reported some fundamental principles of budding in Mediterranean *Tethya *species. During the early stages the stalked buds consist of homogeneous mesenchymal cell masses arranged around a stalk (sclera bundle) rising from the mother sponge. Subsequently, cells migrate into the bud and the mineral skeleton develops. Finally, the bud breaks off, is dispersed by currents and develops into a new sponge. Generally, as long as it is connected to the mother sponge, neither canals nor functional choanocyte chambers form. This means that the adult functions of water pumping and particle uptake are not present at this stage. In some species, however, buds do gain canal system functionality during early development and while still attached to the mother sponge. Buds of *Mycale contarenii *(MARTENS, 1824) are fully functional juvenile sponges which display a notable level of organization and in which all cell types differentiated [20]. The same applies to *Radiospongilla cerebellata *(BOWERBANK, 1863), the only freshwater sponge to display budding [21]. All cases of sponge budding are characterized by the formation of cell aggregates that indicate mesenchymal morphogenesis. In addition, Ereskovsky and co-workers recently reported epithelial budding in the homoscleromorph sponges of the genus *Oscarella *[17,22]. This mode of budding is more similar to budding in cnidarians than the mesenchymal budding of other sponges. However, both mechanisms result in functional clonal juveniles immediately or soon after the release of the buds.

In the present study, we investigated on the basis of the tropical sponge *Tethya wilhelma *SARÀ, SARÀ, NICKEL & BRÜMMER, 2001 (Demospongiae, Hadromerida) whether sponge budding represents a spatiotemporal sequence of morphological events. Adult specimens of *T. wilhelma *typically display a spherical body shape with a distinct outer cortex and an inner choanosome core (see Additional file 1). The cortex is rich in lacuna, while the inner choanosome is characterized by a higher cell density. The architecture of the silica skeleton is strikingly well organized. The most prominent structures of the skeleton are megasclere bundles radiating from the centre, which distally sometimes form small forked fans. Megasters are mainly found in the cortex-choanosome boundary, embedded in strong collagen layers within the mesohyle and forming a megaster sphere around the choanosome. The same applies to micrasters, which form a tylaster layer that is connected to the exopinacoderm [23]. These highly organized parts of the skeleton are made up of biological compound materials (i.e. particle enhanced elastomeres with silica spicules embedded into a collagen/cell matrix) which form functional skeletal superstructures [23,24]. This particular morphological pattern, which is typical of most species of the genus *Tethya*, accounts to the high contractibility of these species [25-27].

Under laboratory conditions *T. wilhelma *seems to reproduces exclusively asexually by budding, but nothing is yet known about reproduction in the wild. Budding occurs all year round, and its frequency and the amount of buds produced vary between individuals. Specific factors influencing budding might be water temperature, salinity or nutrient availability [28]. There seems to be a trend in *T. wilhelma *to intensify budding under changing environmental conditions (unpublished observations). Budding usually starts with the occurrence of tubercles on the surface, which may produce longer filaments that are able to grow into stalked buds [29-31]. It typically takes 48 hours from the moment a developing bud becomes visible on the surface until it is detached from the mother sponge. In the mean time a number of cells and skeletal elements are transported into the bud along the connecting megasclere bundle to form a highly organized almost spherical bud resembling adult morphology [see [23,24]].

We hypothesized that the conspicuous level of organization in *T. wilhelma *develops step by step during bud formation and maturation. We used typical morphological characters like skeleton, canal system and choanoderm as markers to investigate the spatiotemporal patterning of budding. We used synchrotron radiation-based x-ray microtomography (SR-μCT) on complete buds and analyzed the resulting 3D images [23,24,32,33]. In contrast to previous descriptive studies on sponge budding (e.g. [34,20,36,17,18,21]) SR-μCT additionally allows for volumetric analyses. Hence, for the first time, our study addresses morphological changes during bud development in a quantitative manner.

## Results

### Resolution and interpretation of SR-μCT datasets

**Figure 1:**
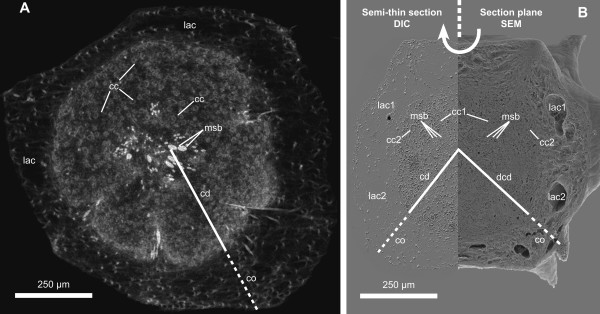
**Morphology of a detached *T. wilhelma *bud (stage 4)**. Comparison between (*A*) a virtual section from an SR-μCT data set and (B) 'combined scanning electron histology' data consisting of a semi-thin section imaged using DIC-microscopy and SEM of the corresponding surface after sectioning (cc - choanocyte chamber, cd - choanoderm, co - cortex, dcd - developing choanoderm, lac - lacunae, msb - megasclere bundle).

Tomographic reconstructions of SR-μCT scans from 11 bud specimens of *T. wilhelma *resulted in high resolution 3D image data in which the morphological structures of complete specimens are visible down to tissue level (see Additional file 2; 5 characteristic data sets are presented, data sets which are not included are available upon request). Image stacks at a voxel size of 1.4·1.4·1.4 μm^3 ^represent a measured resolution of 3.9 μm. Morphological details such as choanocyte chambers were identified, as were the scleres of the highly absorbing silicious skeleton (Fig. 1A). Developing choanosome and cortex structures are clearly distinguishable due to their characteristic cellular density. We validated our morphological interpretation of microtomographic image data by imaging similar buds using SEM and DIC microscopy (Fig. 1B). The resolution of light and electron microscopic imaging methods is higher than in x-ray microtomography, but only the latter provides us with the necessary 3D image data. 3D data facilitate not just visualization, but also volumetrics, providing valuable quantitative information.

### Principle results from qualitative and quantitative SR-μCT image data analysis

**Figure 2:**
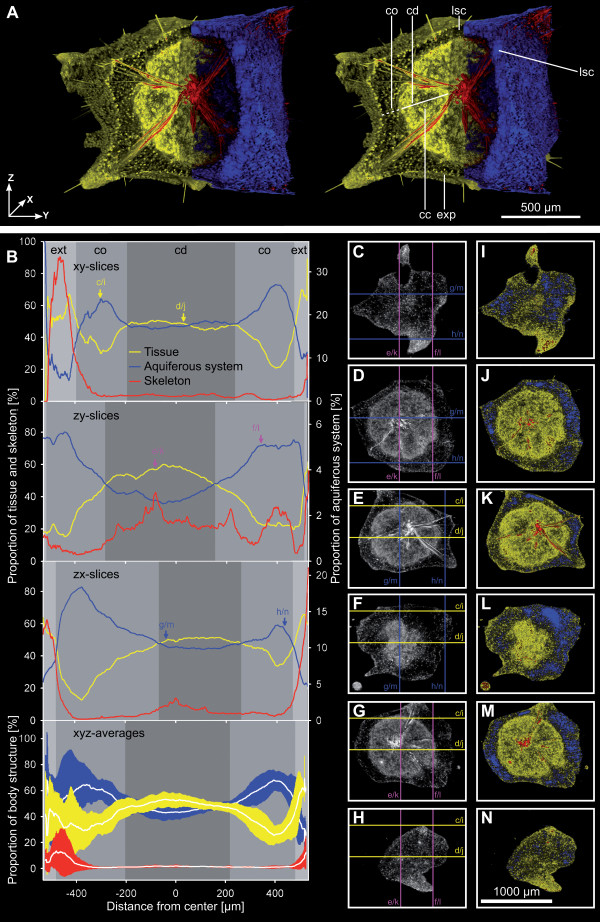
**Detailed visualization and analysis of the overall morphology of a stage 4 *T. wilhelma *bud (data set E, see Additional file **2**) based on SR μCT x-ray absorption and volumetric measurements**. (*A*) Stereo pair rendering with segmentation of morphological structures: sponge tissue (yellow) separated into cortex (co) and choanoderm (cd) with developed choanocyte chambers (cc), exopinacoderm (exp), skeleton (red) and aquiferous system (blue) with lacunar system cavities (lsc). (*B*) Related volumetric measurements. Proportions [%] of sponge tissue, skeleton and aquiferous system measured on 1.4 μm slices. Proportional volume is given for all three spatial directions (x, y and z axes) and as xyz averages with standard deviations in relation to the sponge centre (x, y, z = 0,0,0 μm); arrows and lower case letters refer to C - N (slice images). Main body structures and body extensions (ext) are marked in grayscale. (*C - N*) examples of 1.4 μm slices in grayscale (left column) and colored x-ray absorption-based segmentation of morphological elements (right column). Two slices are presented per dataset direction: xy slices (C, D, I & J), zx slices (E, F, K & L), zy slices (G, H, M & N); lower case letters and lines in grayscale slice images mark the corresponding positions of the orthogonal planes shown as examples in C - N.

In order to present a detailed view of the main results of our qualitative and quantitative data analysis of SR-μCT datasets, this section makes available all the details of one dataset. Since late buds (which are already detached from the mother sponge) display a typical pattern of morphological structures, we have chosen dataset E (see Additional file 2) as an example. Mineral skeleton, aquiferous system and tissue compartment were analyzed. They were distinguished on the basis of their differing x-ray absorption characteristics (Fig. 2). All morphological elements of the sponge could be identified from the volumetric plot. In late buds we found an almost symmetrical arrangement of aquiferous system, tissue and skeleton along the three volume coordinate axes: x axis (= zy-slice stack), y axis (= zx-slice stack) and z axis (= xy-slice stacks) (Fig. 2B). The diameter of the specimen represents ~1000 μm. The coordinate axes are presented relative to the sponge center, thus ranging from -500 μm to +500 μm.

In xy-slices the skeletal proportion varies between 33% and 0.5%, the aquiferous system between 73% and 13% and the tissue between 69% and 22%. The variation of these proportions is not random; it represents the morphological arrangement, which can be checked in the sample slices (Fig. 2C-N): the central choanoderm ranges in size from -199 μm to 236 μm with tissue and the aquiferous system in almost equal proportions. Throughout the sponge, excluding the peripheral regions, the skeletal variation is around 1%. The cortex region itself is situated between -388 μm and -200 μm, and 236 μm and 469 μm, and is dominated by the lacunar cavities of the aquiferous system, with high aquiferous system proportions of up to 63% and 73% and corresponding tissue proportions of 30% and 21% respectively. Body extensions of the sponge are represented in the peripheral regions of the plot outside the cortex region. They are characterized by a reduced aquiferous system and a considerable proportion of skeletal material. As the slim filamentous body extensions are made up of megasclere bundles surrounded by a thin tissue layer, relative values for the skeleton increase significantly in comparison to the body centre.

The same distribution pattern holds for the two additional main axes of the dataset (zy-slice and zx-slice stacks). In the zy-stack, both, cortex (high aquiferous system values) and choanoderm (high tissue values) are identifiable again, with the cortex reaching from -535 μm to - 285 μm and from 160 μm to 506 μm, surrounding the choanoderm (-284 μm to 159 μm) in the centre. The increase in the proportion of skeletal material from 1% to 3% at around - 80 μm indicates the megasclere bundle centre. In the zx-stack the skeletal centre is located around -7 μm: this is indicated by an increase from 1% to 3%. For the cortex and choanoderm the roughly the same pattern is seen as in the former two dataset directions.

By finding the average of the proportions along the three main axes (xyz-averages, Fig. 2B bottom) we obtain a typical pattern. The resulting plots for all three structures are remarkably symmetric to the centre and standard deviations are relatively low. The choanoderm extends -/+ 200 μm from the centre and the cortex affiliates, spreading to a distance of -/+ 480 μm. This is typical of a globular shaped, almost point-symmetrical sponge body resembling the body architecture of an adult *T. wilhelma*. Only in the peripheral parts representing the body extensions the structure proportions are more variable and SD increases.

### Characteristics of distinct bud stages in *T. wilhelma*

Three-dimensional virtual reconstructions and volumetric analysis of 11 μCT data sets of *T. wilhelma *buds revealed a spatiotemporal series for all three functional morphological sponge compartments as exemplified above. Their temporal changes document the development of the sponge cortex and the choanosome into higher level functional morphological units. Depending on the developmental stage of the bud, choanocyte chambers and a network of fine canals are detectable within the choanosome. Many of these canals have diameters close to the resolution limit. The finest canal structures connecting the incurrent system with the choanocyte chambers are thus not resolved in either of the stages.

**Figure 3:**
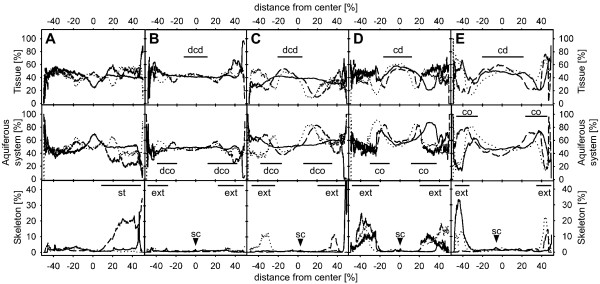
**Volume analysis of body structures in *T. wilhelma *buds based on SR-μCT**. (*A*) stage 1 bud without choanoderm/cortex differentiation; (*B*) stage 2 bud without a separated choanoderm but displaying the first differentiated aquiferous system canals; (*C*) stage 3 bud with an early developing choanoderm (dcd) and developing cortex (dco), (*D *- *E*) stage 4 buds with differentiated choanoderm (cd) and cortex (co) regions. Graphs represent relative volumetric proportions of all main morphological sponge structures: tissue (top row), aquiferous system (middle row) and skeleton (bottom row). Graph patterns typical for distinctly developed sponge regions are marked (sc - skeleton centre, ext - body extension (filaments), st - stalk). Volumetric results are given for the three main axes of the 3D data sets: x-axis (dashed), y-axis (dotted) and z-axis (solid).

The graphs for each bud reveal characteristic spatiotemporal changes in the proportional volumes and quantitative distribution of canals, tissue and skeleton within the buds (Fig. 3). The overall graph shape for each structure changes specifically during bud formation and maturation and displays characteristic features at each stage. Newly formed buds display a homogenous distribution of tissue and aquiferous system elements (Fig. 3A). Step by step, the morphology changes into the adult phenotype, which is characterized by a high proportion of tissue in the sponge centre (the choanosome) and a high proportion of peripheral aquiferous system lacunae (the cortex), and by mineral scleres in the filamentous body extensions protruding from the sponge surface. As shown above, in juveniles which are fully functionally developed (late bud stages), all structures are represented by distinct peaks or plateaus. However, the cortex and choanosome also leave an imprint on the graphs in their early developmental stages (Fig. 3B-C).

Buds fall into four categories which we defined by combining volumetrics, graph characteristics and qualitative characters (i.e. overall shape of the bud, skeleton organization, differentiation of cortex and choanosome, presence and organization of large canals). The resulting spatiotemporal morphological pattern sequence is typical of bud formation and maturation.

### Stage 1 buds

**Figure 4:**
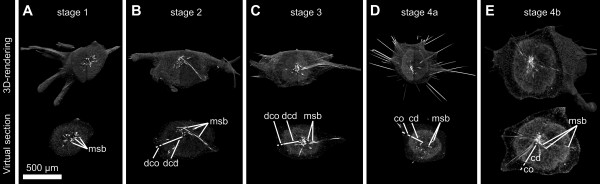
**3D volume renderings of stage 1 to 4 buds of *T. wilhelma *and corresponding virtual sections from SR-μCT data elucidating the development of distinct morphological structures**. (*A*) stage 1 bud without choanoderm/cortex differentiation (msb - megasclere bundle); (*B*) stage 2 bud without a separated choanoderm (dcd - developing choanoderm, dco - developing cortex) but displaying the first differentiated aquiferous system canals; (*C*) stage 3 bud with an early developing choanoderm (dcd) and developing cortex (dco), (*D *- *E*) stage 4 buds with differentiated choanoderm (cd) and cortex (co) regions, the latter displaying lacunar cavities.

**Figure 5:**
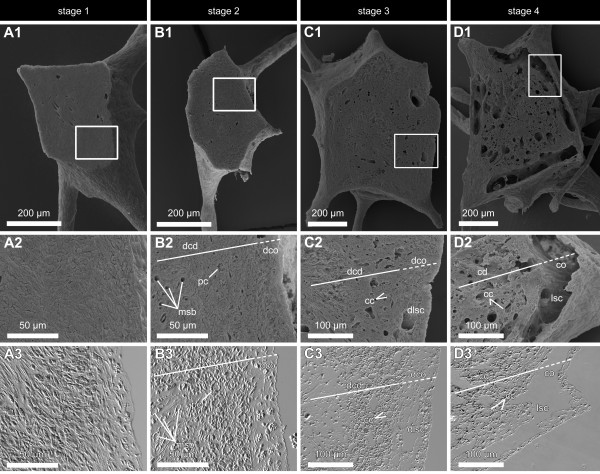
**Corresponding SEM and DIC images of stage 1 to stage 4 buds of *T. wilhelma *A1 - D1**. SEM images of median section planes of the four bud stages of *T. wilhelma*, Framed areas are represented in A2 - D2 (SEM) and the DIC microimages of corresponding semi-thin sections in A3 - D3. In stage 1 buds (A) cells are densely packed and evenly distributed. Stage 2 buds (B) display early developing cortex (dco) and choanoderm (dcd) regions as well as few numbers of primordial choanocytes (pc) and megasclere bundles (msb). Developing lacunar system cavities (dlsc) are found in stage 3 (C) as separated developing choanoderm and cortex regions. In stage 4 (D) clearly developed lacunar system cavities (lsc), choanocyte chambers (cc) and distinguished cortex (co) and choanoderm (cd) are present.

Stage 1 buds exhibit a homogenous distribution of tissue and aquiferous system components (Figs. 3A). Cortex and choanosome are not differentiated, neither are lacunar cavities and choanocyte chambers (Figs 3A, 4A, 5A, and see Additional file 3A). The proportional volume of the skeleton is almost constant at around 1.5% throughout the complete specimen. In the stalk that connects the bud with the mother sponge, however, the proportional skeletal volume increases to over 30% (see Additional file 3A, arrowhead). The megasclere bundle of the stalk defines the main axis of the bud skeleton and consequently the whole bud at this stage. The overall appearance of the bud at this stage is flat and elongated along its axis (Fig. 4A, see Additional file 4). Apart from the stalk, a few megasclere bundles start to form around the future centre of the skeleton. All the bundles are arranged in a plane perpendicular to the stalk (see Additional files 3A and 4).

### Stage 2 buds

Stage 2 buds are still connected to the mother sponge through the stalk (see Additional file 3B) but are more globular in shape and display more advanced skeletal development (Fig. 4B). The megasclere bundles radiate into additional directions, but the bundles and the skeletal centre are not as highly organized as in the adult skeleton. The skeleton is more planar star shaped than globular. The cortex and choanosome regions are not yet differentiated (Figs. 3B, 5B, and see Additional file 3B), but a slight increase in the aquiferous system concurrent with a decrease in the tissue at relative distances greater than 20% from the centre indicate that the differentiation of tissue and aquiferous system has already started (Fig 3B). This process is accompanied by the first occurrence of a small number of choanocyte chambers. The bud still appears flattened as a consequence of the planar arrangement of the skeleton (Fig. 4B)

### Stage 3 buds

Stage 3 buds start to develop distinct aquiferous system lacunae in the cortex region and a denser choanosome around the centre (Figs. 4C, 5C, and see Additional file 3C). Accordingly, volumetric graphs show their first peaks for both structures (Fig. 3C). Visual analysis of 2D image slices and volume renderings indicate the presence of choanocyte chambers in the developing choanosome (Fig. 5C). The skeleton centre is prominent, focusing on a single point (see Additional file 3C). This is also represented in the volumetric graphs by small central peaks (Fig. 3C). The skeleton is almost star shaped in 3D, but still retains a certain flatness which also characterizes the outer shape (Fig. 4C, and see Additional file 3C). Stage 3 buds are still connected to the mother sponge through the initial stalk.

### Stage 4 buds (juveniles detached from mother sponge)

Stage 4 buds display an adult-like body structure (Figs. 4D-E, 5D, and see Additional file 1 and Additional file 3D-E). The dense choanosome core stands out against the peripheral cortex, which has prominent lacunar system cavities. Within the choanosome, differentiated choanocyte chambers are present in high numbers (Figs. 1A, 5D). Volumetrics graphs show an almost symmetrical pattern for the two body compartments (Fig. 3D-E). This almost globular graph pattern and outer shape (Figs. 4D-E, and see Additional files 3D-E and Additional file 5) is a consequence of the way in which the main skeleton is arranged. The number of megascleres has increased, and the bundles are arranged homogenously, forming a spherical star (see Additional file 3D-E and Additional file 4). We found two different cases which we regard as subtypes of the same stage (represented by D and E in Figs. 3, 4, and in Additional file 3; see also Additional file 6). In both cases the buds were detached from the mother sponge. The main difference is the total volume, which is affected by the proportion of tissue and skeleton (see Additional file 6). It is not possible in either case to identify the site of the former connecting stalk, as its megascleres have been integrated into the main skeleton (see Additional files 3D-E and Additional file 5). Stage 4b, the more advanced of the two, is characterized by a higher cell mass, a higher number of megascleres per bundle and a number of finer megascleres which are apparently synthesized within the bud (Fig. 4E and see Additional file 3E). This particular stage represents a juvenile sponge and seems to be fully functional. In contrast, the earlier stage 4a displays lower biomass and less prominent megasclere bundles (Fig. 4D and see Additional file 3D). On the other hand, the aquiferous system is equal to the one in stage 4b, and thus in the adult sponge (Figs 3D-E, 4D-E). Interestingly, the diameter of stage 4a buds is smaller and the biomass is lower than in the examples for stages 1 to 3. The developmental stage of a bud, then, cannot be inferred directly from its diameter.

## Discussion

### Methodology

A number of morphological methods are available for studying temporal morphological developmental series. Synchrotron radiation-based x-ray microtomography (SR-μCT) has the advantage of offering detailed access to general 3D-morphology without the need to section specimens [24,33]. In the case of sponges, morphological structures such as skeletal elements (spicules) can be viewed in their undisturbed context [23]. Classical histological semi-thin sections and light microscopic analysis give higher resolutions, but the sectioning itself destroys spicules (if they have not had to be dissolved beforehand). However, μCT should not be treated as a substitute for microscopic methods, but as a way of broadening the range of methodological tools available [24,37]. Another benefit is that μCT is not limited to visualization. It allows morphological patterns to be identified and quantified using 3D-morphometrics [24]. This turned out to be an important prerequisite in defining discrete bud stages of *T. wilhelma*. SR-μCT-based volumetrics of the main morphological elements, i.e. the aquiferous system, tissue and skeleton, provided information that was essential in establishing a sequential bud series. Indeed, μCT allowed us to follow poriferan asexual reproduction quantitatively for the first time ever.

### Spatiotemporal morphological patterning during budding in *T. wilhelma*

Poriferan budding is a key element in understanding the evolution of asexual reproduction in metazoans. The morphogenesis that takes place during bud development in *T. wilhelma *passes through a spatiotemporal series of four distinct bud stages. Thus, sponge asexual developmental processes are spatiotemporally patterned, comparable to budding in cnidarians [e.g. [38,39]]. What conclusions can we draw from our results in terms of a general interpretation of sponge budding? And in a wider context, what can we learn about the early evolution of asexual reproduction and its regulation in Metazoa?

**Figure 6:**
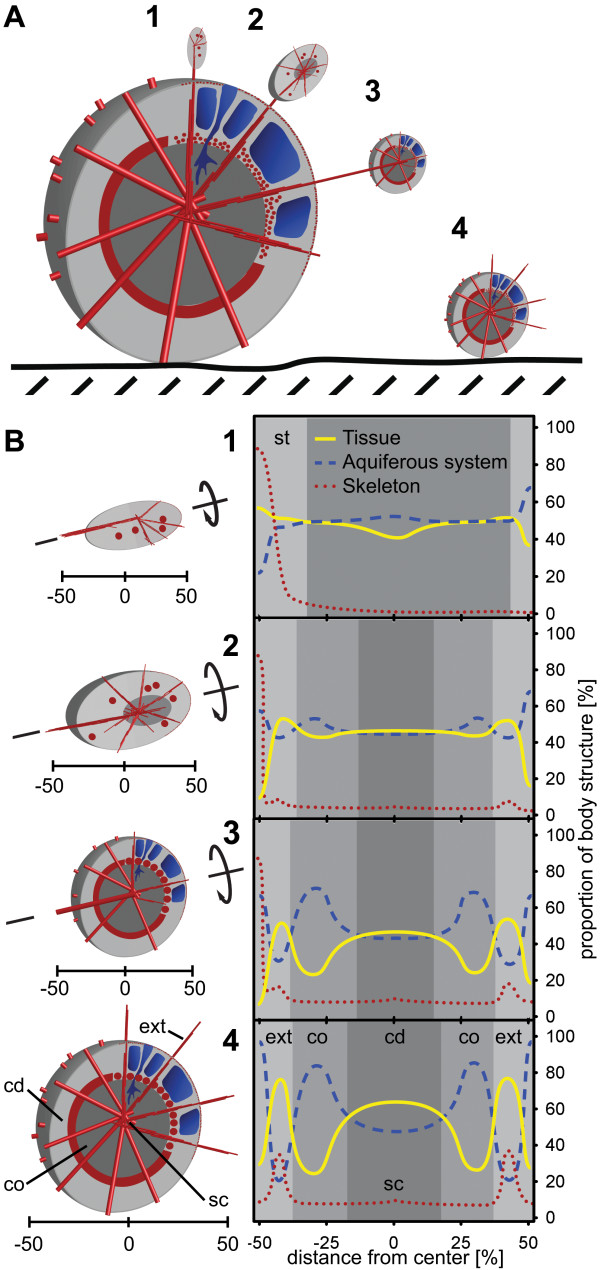
**Scheme of bud development in *T. wilhelma***. (*A*) Four bud stages are characteristic, with the first three connected to the mother sponge by a stalk: Skeletal elements in red (megasclere bundles and aster spheres); megasclere bundles partly simplified as cylinders; Tissue in grey, separated into cortex (light grey) and choanoderm (dark grey). (*B*) Details of bud stages (left) and schematic graphs of morphological functional unit distribution. There are indications of rotational symmetry along the initial connecting stalk (st) in stages one to three (compare Additional file 4). Stage 4 buds display an adult-like body morphology with point symmetry to the skeleton centre (sc; see Additional file 5). Choanoderm development starts in stage 2, accompanied by the development of the megaster spheres in stage 3. Differentiation into a cortex (co) and choanoderm (cd) is characterized by the development of the aquiferous system (larger canals in stage 2; lacunae in stage 3). Body extensions (ext) (filaments) are found in stage 4 buds. For further details see text.

Assuming development is continuous, the distinct stages in bud development in *T. wilhelma *represent important milestones of bud morphogenesis. The morphological changes are schematically summarized in Figure 6. Budding starts with the migration of cells and the transportation of the first megascleres into the early bud [not investigated here, for details see [31]]. Stage 1 buds are dominated by the stalk connecting the bud and the mother sponge, which also represents a first symmetry axis (Fig. 6B1, see Additional file 4). Cells migrating into the emerging bud from the mother sponge arrange axisymmetrically around the tip of the stalk, forming a characteristic small bulb. The future skeletal centre is formed within the centre of this cellular bulb (Fig. 6B2). The overall spatiotemporal pattern of bud morphological development is characterized by several temporally overlapping processes: 1. Rearrangement of megascleres from the primary axis via a planar star to a spherical star shape. 2. Formation of the aquiferous system, constituted by choanocyte chamber differentiation (compare Fig. 1) and canal formation. 3. Differentiation of a choanosomal centre and a cortical region, which seems not to start before the skeleton merges from the planar star to the spherical star shape (Fig. 6B3-4; compare Figs. 3B-C and see Additional file 3B-C). This process coincides in most cases with the release from the mother sponge, and we regard it as the onset of the full functionality of the aquiferous system. A comparison between bud sizes and the proportion of skeletal elements to tissue reveals no correlation (see Additional file 2), so bud size does not seem to be significant in the spatiotemporal pattern of bud development.

Our results demonstrate that choanosome and cortex develop in correlation with the differentiation of the star shaped skeleton. This is documented from stage 3 onwards and is crucial in overall bud morphogenesis. The importance of skeletal development in bud formation has been hypothesized previously [18], but the present 3D morphometric analysis makes it possible to quantify the link between skeleton arrangement and cortex differentiation (see Fig. 3). In the detached juvenile sponges (stage 4 buds) lacunae and a wide canal system are already highly developed, as in adult specimens. A high number of choanocyte chambers are also found. These juvenile sponges can be regarded as morphological and functional equivalents of adult *T. wilhelm*a specimens (Fig. 6B4). By this stage, the series of events that play a role in bud development has been completed. Apart from general body growth, which takes place by means of spicule synthesis, cell division, differentiation and extracellular matrix production, no further changes in morphological patterning occur. From a structural point of view a steady state has been reached which indicates that the juvenile sponge has developed adult-like body architecture. From now on it will only increase in size. This may involve a substantial volume increase of up to 7.500× or above. Larger adults with a diameter of approximately 2 cm typically have a volume of around 4.2 cm^3^. We assume that in stage 4 the sponge reaches the state of 'constant morphogenesis' [12].

### Budding in other demosponges

Asexual reproduction processes in demosponges have been studied previously, especially budding in *Tethya maza *SELENKA, 1879 by Selenka himself [40] and in *Tethya lyncurium *(PALLAS, 1766) by Maas [18] and Connes [19,35] (Remark: *T. lyncurium *is an unaccepted junior synonym of *T. aurantium *(PALLAS, 1766); Eventually one or both authors worked on *T. citrina *SARÀ & MELONE 1965, which was described as a cryptic species formally addressed as *T. aurantium/T. lyncurium*, too). In this species the development of the skeleton is seen as a key element in the budding process. Just as in *T. wilhelma *the initial megascleres are originating from the mother sponge, forming the center of the new bud. Soon spicule production starts in the emerging bud too. For early buds a fan-shaped configuration of the skeleton has been described which later changes to a radial star shaped architecture like in our case. Maas for the first time differentiated stages in bud development. We regard his 'fan-shaped bud stage' of *T. lyncurium *to represent the same developmental level than our 'stage 1' buds of *T. wilhelma*. A similar stage 1 bud has been illustrated in *Tethya seychellensis *(WRIGHT, 1881) by Sollas [41]. In *T. lyncurium *the formation of larger distinct megasclere bundles and the radial star shaped configuration of the bundles typically organize after the release from the mother sponge. In contrast, we found the similar organizational level in stage 3 buds of *T. wilhelma*, which are still attached to the mother sponge. For buds just about to be released Maas observed the onset of choanoderm and cortex separation. This corresponds to our definition of a stage 2 bud. In direct comparison, distinct morphological structures develop earlier in *T. wilhelma *than in *T. lyncurium *The findings by Maas are supported by Connes who notes that the buds at their release show distinguishable regions which remind of adult cortex and choanoderm but still differ from the adult micromorphology [19,35]. Newly released buds for example lack lacunae, canals and choanocyte chambers. In both studies it remained unclear whether canals or choanocyte chambers are to be formed first. In contrast to *T. wilhelma *which releases fully functional juvenile sponges after a considerably short period of 2 days in *T. lyncurium *the development takes weeks to month. Similar observations have been reported for *T. aurantium *und *T. citrina *[42,43]. Buds are released without a functional aquiferous system and would correlate to *T. wilhelma *stage 2 or 3. For early bud stages the authors also describe densely packed cells and suggest the skeleton as an important factor in bud development. In all cases bud size is no indicator for the developmental stage of the newly forming sponge.

In *Axinella damicornis *(ESPER, 1794) external buds are derived from the ectosome and display a similar cellular composition [34]. This is similar to *T. aurantium *and *T. citrina*. Additionally, all three species share the lack of an aquiferous system. However, no skeletal elements are present in buds of *A. damicornis*. The developmental sequence of budding in this species remains to be studied in detail.

In contrast, buds of *T. maza *posses choanocyte chambers and canals in the developing choanoderm [40]. In addition, the cortical system of lacunar cavities develops prior to the release from the mother sponge. Nevertheless, the lacunar cavities and the aquiferous system of the developing choanoderm are not connected at this point. Such buds in *T. maza *seem to be similar to stage 3 buds in *T. wilhelma*. Apart from *T. maza *and *T. wilhelma*, the release of fully functional buds has been reported only for *M. contarenii *[20,36]. In this species a well developed aquiferous system is present in newly detached buds. In young buds the cells are distributed evenly, in some cases grouped together in a more compact configuration, in others packed more lose. With the ongoing development choanocytes converge to form chambers along the canals. This process is accompanied by the formation of lacunae and the separation of a choanosome. Therefore the released buds of *M. contarenii *could be compared to stage 4 buds in *T. wilhelma*. In all investigated demosponge species distinct patterns in bud development were found. If these patterns are compared among different species there are characteristic patterns occurring in all species only differing on the development time scale and order (heterochrony).

## Conclusion

### Is budding in sponges generally following characteristic spatiotemporal patterns?

This study raises the question of whether the series of morphological changes observed are specific to *T. wilhelma *or a general phenomenon in sponges. The remarkable degree of variation in budding processes in sponges, in terms of the time scale governing body structure development and differentiation and the developmental stage which has been reached at detachment, is a sign of general heterochrony. Bud development can take from 24 hours (*R. cerebellata*) to several months (*T. lyncurium*) [19,21] in total, with the time between the different stages also varying considerably. Some species release their buds as fully functional individuals [20,21] corresponding to *T. wilhelma *stage 4 buds. In contrast, some species release buds that are organized parenchymally [19,43], resembling the level of tissue organization of a parenchymula larva. These buds lack functional elements of the aquiferous system, such as choanocyte chambers or canals, at the point of their detachment, and start their final phase of development and differentiation after having settled to the substratum [17]. Their organizational level at release is similar to that of *T. wilhelma *stage 2 buds. Despite the effects of this heterochrony, a re-evaluation of published data reveals similar spatiotemporal patterns in all *Tethya *species [18,19], *Mycale *species [20], *R. cerebellata *[21] and even the homoscleromorph sponges [17]. This indicates that spatiotemporal morphogenetic pattern sequences are common phenomena in the asexual reproduction of poriferans. From the present data and published examples of sponge budding we conclude that the most highly organized sponge morphologies generally occur in late buds/early juveniles (detached). In some sponges, e.g. *R. cerebellata*, overall body morphology even seems to be more highly organized than later adult stages (compare Fig. 2 by Saller [21]).

### Morphogenetic processes during asexual reproduction

Development from the earliest stages to complex buds requires, over a certain time period, differentiation and a number of distinct and directed cell movements, e.g. the specific arrangement of spicules mediated by specialized cells such as sclerocytes. Such spatiotemporally defined morphogenetic processes are similar to morphogenetic cell movements during embryonic and larval development in Eumetazoa. In various phyla, for instance, gastrulation is characterized by ingression and cell immigration which lead to distinct morphological patterns [44]. In a similar biomechanical manner, budding is characterized by ingression and the immigration of autonomous mesenchymal cells from the mother sponge. This is referred to as mesenchymal morphogenesis and is distinct from the epithelial morphogenesis that takes place during budding in Cnidaria and also homoscleromorph sponges [17,22]. However, pinacoderm eversions also play a role in budding dominated by mesenchymal morphogenesis, at least in the very first phase of bud formation.

Morphogenetic processes in Metazoa are generally regulated by developmental genes. Recent results of genome sequencing projects have demonstrated the presence of a variety of developmental genes in all metazoan phyla [45], including poriferans [46-48]. Members of the *Wnt *gene family, for example, are expressed in embryos and are involved in establishing axial polarity, e.g. in cnidarians [49,50]. *Wnt *is also expressed in the larvae of the sponge *Amphimedon queenslandica *HOOPER & VAN SOEST, 2006 (aka. *Reniera *sp.) in the context of anterior-posterior polarity [46]. In cnidarians, these developmental pathways are not limited to larval morphogenesis. In *Hydra*, for example, *HyWnt *plays a central role in the formation and maintenance of the head organizer and in axis formation in new buds [10]. Similar effects have recently been demonstrated in adult poriferans, where *Wnt *is involved in the formation of ostia [48]. It is likely that future studies will reveal that *Wnt *is also involved in morphological pattern formation during bud development in sponges. However, this will have to be proven experimentally.

In order to further investigate developmental processes model sponge systems are required for experimental purposes. No sexually reproducing species can currently be maintained in closed laboratory systems under controlled conditions. In contrast, the asexual reproduction cycle of *T. wilhelma *is complete which allows this species to be reared and kept under laboratory conditions all year round. Distinct bud stages can be collected and kept under controlled conditions. This makes *T. wilhelma *an ideal model system for this kind of study. As demonstrated in cnidarians, basal, genetically regulated developmental processes present in larval development also control asexual reproduction. In order to understand the early evolution of asexual reproduction in metazoans it will be crucial to investigate the regulation of spatiotemporal patterning in sponge budding and bud development. Moreover, investigations of this nature will complement similar studies on larval development following sexual reproduction currently being carried out [46,47].

## Methods

### Animals and culturing

Specimens of *T. wilhelma *were initially sampled in the zoological-botanical garden Wilhelma, Stuttgart, Germany and cultured for years in laboratory aquariums [30]. Buds of varying ages/sizes were collected from budding adult specimens.

### Synchrotron radiation based X-ray computed microtomography

Buds were shock frozen in liquid nitrogen immediately after sampling and subsequently freeze substituted in a Leica AFS with methanol + 1% OsO_4 _+ 2.5% glutaraldehyde + 2.5% distilled water [modified after [24]]. They were transferred to acetone, critical point dried in an Emitech K850 critical point drier and glued to a specimen holder.

SR-μCT was performed at beamline BW2 of HASYLAB (Hamburger Synchrotronstrahlungslabor) at the Deutsches Elektronen-Synchrotron DESY [32], using monochromatic X-rays at a photon energy of 11 keV. Radiograms (n = 720) were taken equally stepped between 0 and π (average exposure time of 9.5 s, for details see Additional file 2). Stacks of ≥ 1,024 images were calculated from each set of radiograms using the tomographic reconstruction algorithm of 'back projection of filtered projections' [51].

### Image data sets

Original SR-μCT raw image data were converted to uncompressed 8-bit Bitmap stacks by segmenting grey value information from the most informative 32-bit floating point raw data. Image processing included the following steps: 1. Conversion of raw data to 8-bit image data, 2. 3D adaptive Gauss filtering, 3. Creating and correcting a mask for voxels outside the sponge body, 4. Subtracting the mask from the 8-bit image data to create a virtual cast of the canal system and permit volume measurements. All image processing steps were logged. All original and intermediate data sets were archived. We used ImageJ for image conversion and processing, including image inversions, artifact correction, filtering, thresholding and creation of 2-bit masks. Additional filtering of image stacks was carried out using VGStudioMax (Version 2.0, Volume Graphics GmbH) and applying a 3D adaptive Gauss filter.

### Volume rendering

Sponges and their internal morphological elements (skeleton, aquiferous system and tissue) were 3D-rendered from reconstructed image stacks using VGStudioMax (64-bit Version 2.0, Volume Graphics GmbH) applying the volume rendering algorithm "Scatter HQ". Segmentation and visualization of all morphological elements was based on grey value thresholding. 3D images were rendered at full resolution (1.4 μm voxel size)

### Quantitative image analysis

Volume measurements were performed on processed, segmented image stacks in ImageJ, using similar classifications to those used in rendering. For each morphological functional element three classifications were used and means were calculated in order to reduce classification errors. Volumes were calculated by adding together the volume measurements of all stack images. Volumes were calculated three times independently along each image volume axis to average out masking errors [24]. Structure volumes were plotted as a proportional share of total bud volume as a function of the distance from the calculated bud centre. In order to compare different sized buds, we used relative distances from bud centers.

### Scanning electron microscopy histology

We applied correlated imaging to samples viewed in SEM and DIC microscopy [52]. Buds were fixed in a 0.45 M sodium acetate buffer (pH 6.4 in filtered aquarium seawater) + 2% OsO_4 _+ 2% glutaraldehyde + 0.29 M sucrose immediately after sampling. They were desilified in 5% hydrofluoric acid for 1 h and then embedded in styrene-methacrylate [52]. After semi-thin sectioning, we dissolved the plastic around the remaining sponge part using xylene-treatment and dehydrated the samples in increasing concentrations of acetone. Specimens were critically point dried in an Emitech K850 CPD system and sputter coated in an Emitech K500 SC system. SEM images were taken on a Philips XL30ESEM.

## Competing interests

The authors declare that they have no competing interests.

## Authors' contributions

MN and JUH designed research; all authors performed SR μCT experiments; MN and JUH performed SEM/DIC histology; FB and JH reconstructed tomographic image data; JUH and MN analyzed and rendered image data, prepared the figures and wrote the paper. All authors revised the paper.

## Supplementary Material

Additional file 1 **Habitus, principal morphology and budding behavior of *T. wilhelma***. Figure: (*A*) Schematic morphology (left) and habitus (right) of an adult specimen of *T. wilhelma*. Elements of the mineral skeleton are colored in blue, collagen layer in light blue, tissue in grey, canals and lacunae in white (cbs - collagen boundary sphere; cd - choanoderm; co - cortex; ec - excurrent canal; exp - exopinacoderm; ext - body extension (filaments); lsc - lacunar system cavities; mas - megaster sphere; msb - megasclere bundles; msbt - megasclere bundle tip; tal - tylaster (microscleres) layer). (*B*) Budding specimen of *T. wilhelma *with stage 1 to 3 buds characteristically still connected to the mother sponge, and detached stage 4 buds. Click here for file

Additional file 2 **Beamline settings for SR μCT**. Table: Experimental settings at DESY-beamline HASYLAB BW2 for synchrotron radiation based x-ray microtomography of *T. wilhelma *buds. Click here for file

Additional file 3 **Grouped projections of *T. wilhelma *buds from SR-μCT data**. Figure: x, y & z projections according to the data set directions representing four different developmental stages: (*A*) stage 1, (*B*) stage 2, (*C*) stage 3, (*D*) stage 4a and (*E*) stage 4b. The development of the highly absorbing skeletal elements can be followed from the early stages, when only one main symmetrical axis is present, defined by the stalk (arrowheads), to the later stages, when the megasclere bundles and the developing megaster sphere are arranged point symmetrically. From stage 3 onwards (C) the developing choanoderm starts to be distinguishable and can be followed to the fully differentiated choanoderm in stage 4 (E). Click here for file

Additional file 4 **3D-morphology of a stage 1 bud**. Movie: 360° rotation of projections (left) and a volume rendering (right) of microtomographic data. For details refer to text and figures 4. Click here for file

Additional file 5 **3D-morphology of a stage 4 bud**. Movie: 360° rotation of projections (left) and a volume rendering (right) of microtomographic data. For details refer to text and figures 4. Click here for file

Additional file 6 **Volumetric data for bud compartments**. Table: Comparison of size, volumes and proportional volumes (%) of mineral skeleton, tissue and aquiferous system in developing buds of *T. wilhelma*. Click here for file
