# Supplementary figures and images for: Sponge budding is a spatiotemporal morphological patterning process: Insights from synchrotron radiation-based x-ray microtomography into the asexual reproduction of Tethya wilhelma
Source: Front Zool. 2009 Sep 8;6:19. doi: 10.1186/1742-9994-6-19 (PMC2749020; doi:10.1186/1742-9994-6-19)

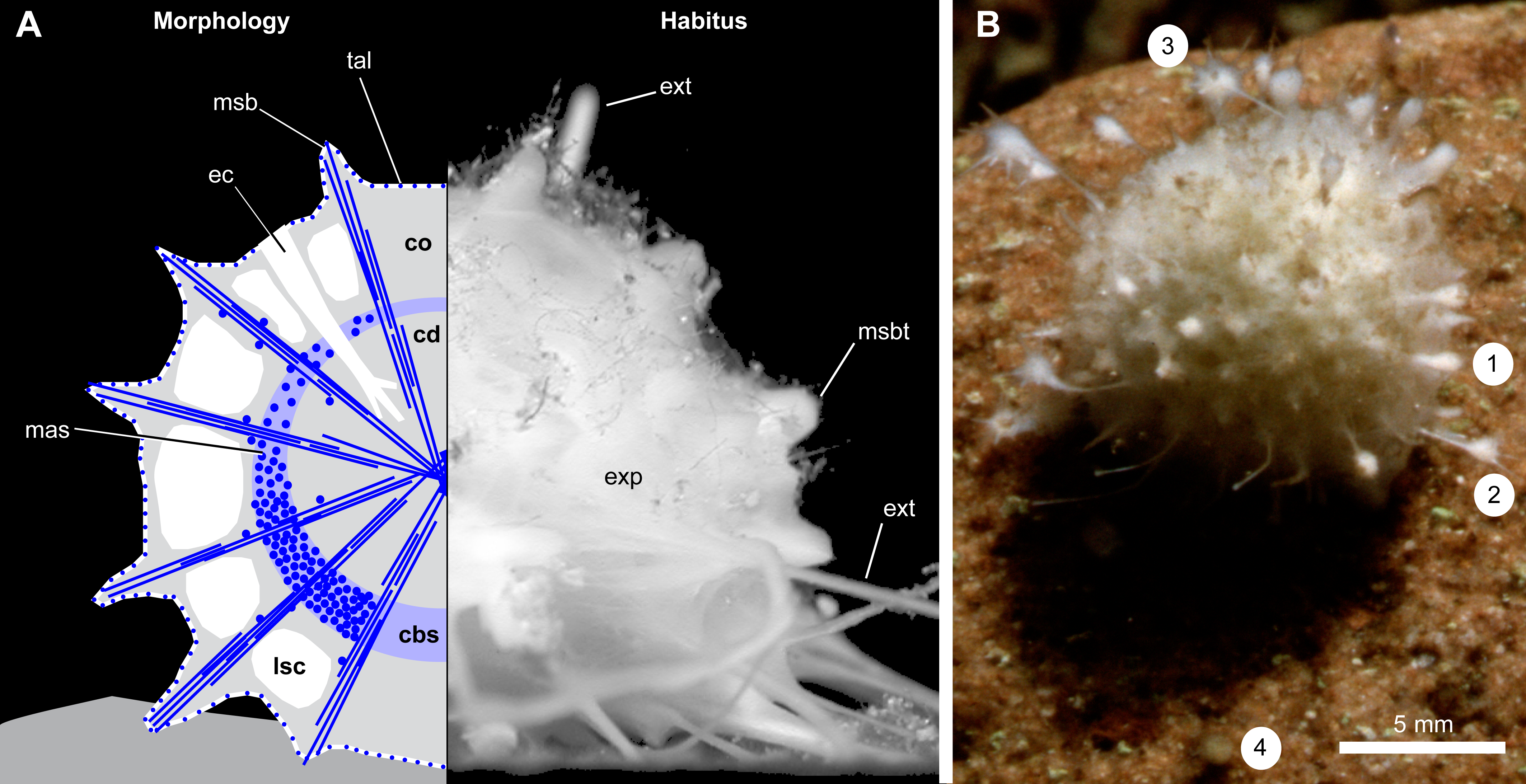

Supplement: Additional file 1 — Habitus, principal morphology and budding behavior of T. wilhelma. Figure: (A) Schematic morphology (left) and habitus (right) of an adult specimen of T. wilhelma. Elements of the mineral skeleton are colored in blue, collagen layer in light blue, tissue in grey, canals and lacunae in white (cbs - collagen boundary sphere; cd - choanoderm; co - cortex; ec - excurrent canal; exp - exopinacoderm; ext - body extension (filaments); lsc - lacunar system cavities; mas - megaster sphere; msb - megasclere bundles; msbt - megasclere bundle tip; tal - tylaster (microscleres) layer). (B) Budding specimen of T. wilhelma with stage 1 to 3 buds characteristically still connected to the mother sponge, and detached stage 4 buds. [file 1742-9994-6-19-S1.tiff]
