# Supplementary material for: Sponge budding is a spatiotemporal morphological patterning process: Insights from synchrotron radiation-based x-ray microtomography into the asexual reproduction of Tethya wilhelma
Source: Front Zool. 2009 Sep 8;6:19. doi: 10.1186/1742-9994-6-19 (PMC2749020; doi:10.1186/1742-9994-6-19)
Supplement: Additional file 2 — Beamline settings for SR μCT. Table: Experimental settings at DESY-beamline HASYLAB BW2 for synchrotron radiation based x-ray microtomography of T. wilhelma buds. [file 1742-9994-6-19-S2.pdf]

Supplementary Table 1

Tab. S1. Experimental settings at DESY-beamline HASYLAB BW2 for synchrotron radiation-based x-ray microtomography of *T. wilhelma* buds.

Sample (*T. wilhelma*)	A	B	C	D	E
Dataset identifier	2007h-usb08a	2007h-usb03a	2007h-usb06a	2007h-usb07a	2007h-usb02a
xyz-dimension (voxels)	1536·1536·690	1536·1536·409	1536·1536·334	1536·1536·631	1536·1536·764
xy-pixelsize (µm/pixel)	1.4	1.4	1.4	1.4	1.4
z-spacing (µm)	1.4	1.4	1.4	1.4	1.4
Resolution (µm)	3.9	3.9	3.9	3.9	3.9
Photon energy (keV)	11	11	11	11	11
Exposure time (s)	9.5	10	9.4	9.5	9
