# Supplementary material for: Sponge budding is a spatiotemporal morphological patterning process: Insights from synchrotron radiation-based x-ray microtomography into the asexual reproduction of Tethya wilhelma
Source: Front Zool. 2009 Sep 8;6:19. doi: 10.1186/1742-9994-6-19 (PMC2749020; doi:10.1186/1742-9994-6-19)
Supplement: Additional file 6 — Volumetric data for bud compartments. Table: Comparison of size, volumes and proportional volumes (%) of mineral skeleton, tissue and aquiferous system in developing buds of T. wilhelma. [file 1742-9994-6-19-S6.pdf]

Supplementary Table 2

Tab. S2. Comparison of size, volumes and proportional volumes (%) of mineral skeleton, tissue and aquiferous system in developing buds of *T. wilhelma*.

Specimen	Size [µm · µm · µm]	Volume [mm^3]	Skeleton [%]	Tissue [%]	Aquiferous System [%]	Unassigned* [%]	Dataset identifier
A	506 · 532 · 441	0.08	1.7	41.0	51.1	6.2	2007h-usb08a
B	862 · 815 · 534	0.21	0.7	42.0	48.4	8.9	2007h-usb03a
C	699 · 671 · 449	0.13	0.6	37.7	53.4	8.3	2007h-usb06a
D	547 · 572 · 502	0.09	1.0	38.4	59.0	1.6	2007h-usb07a
E	1111 · 1008 · 922	0.55	1.5	46.9	51.1	0.5	2007h-usb02a

***** "Unassigned" data: a small number of voxels within the buds could not be definitely assigned to any one of the morphological functional units.
